# Editorial: The Maintenance of Genome Integrity in Plants: Novel Challenges in Basic and Applied Research

**DOI:** 10.3389/fpls.2020.00585

**Published:** 2020-05-15

**Authors:** Alma Balestrazzi, Kaoru O. Yoshiyama, Ayako N. Sakamoto

**Affiliations:** ^1^Department of Biology and Biotechnology “L. Spallanzani”, University of Pavia, Pavia, Italy; ^2^Life Sciences, Tohoku University Aoba-ku, Sendai, Japan; ^3^Department of Radiation-Applied Biology Research, National Institutes for Quantum and Radiological Science and Technology, Takasaki, Japan

**Keywords:** genome integrity, DNA repair, DNA damage, UV, double strand breaks, cell cycle

Plants are sessile organisms endowed with astonishing genomic plasticity. A plethora of undesired events challenge DNA integrity and a deeper knowledge of the mechanisms underlying DNA repair and maintenance of genome stability will help improving our understanding of how plants cope with hostile environments in this era of global climate change. This Research Topic provides a comprehensive overview of the current progress on the study of the genotoxic stress response in plants at the cellular and molecular level.

Roldán-Arjona et al. provide an exhaustive review of the recent advances in the study of base excision repair (BER). Plants share several BER factors with other organisms, although possessing unique features elucidated by biochemical and genetic studies. The review underlines the gap of knowledge regarding the identification of DNA polymerases involved in gap filling, the interplay between BER factors and chromatin remodeling mechanisms, and the BER pathways within mitochondria/chloroplasts. Similarly, Sakamoto et al. reports on the state of the art of translesion synthesis (TLS), one of the pathways used to overcome stalled replication. TLS polymerases are generally low-fidelity enzymes, prone to induce mutations. The plant DNA polymerase ζ, η, κ, θ, and λ and Reversionless1 (Rev1), involved in the TLS, have been studied at the genetic level using mutants which display enhanced sensitivity to DNA-damaging agents. There is evidence that TLS polymerases act in parallel with the Rad5-dependent pathway involved in the repair of the stalled replication fork. Current studies point at plants as an ideal model for assessing the role, regulation, and interaction of TLS polymerases since, differently from animals, these functions can be disrupted in plants without severe reduction of fertility.

The contribution of NER (nucleotide excision repair) to the removal of UV-induced DNA lesions has been clarified by Al Khateeb et al.. They showed in *Arabidopsis thaliana* that loss of function mutants of *AtUVSSA* (*UV Stimulated Scaffold protein A*), *AtUSP7* (*Ubiquitin Specific Peptidase 7*), and *AtTFIIS* (*RDO2, Reduced Dormancy 2*) genes exhibit increased UV sensitivity. This finding highlights the conserved role of such NER components in the DNA damage response (DDR) triggered by UV radiation.

The increasing number of studies dealing with DNA damage accumulated in the embryo genome and the repair capacity of the seed has been extensively reviewed by Waterworth et al.. These authors underline how DDR factors participating in genome maintenance represent promising targets for the genetic improvement of crop germination performance in the field, in particular under stress conditions.

Meiotic DNA recombination requires crossing-over and chromosome segregation. Crossing-over begins with double-strand breaks (DSBs) induction. Disrupted Meiotic cDNA1 (DMC1) is a conserved recombinase that searches for and invades homologous sequences to aid in the repair of meiotic DSBs. Szurman-Zubrzycka, Baran et al. identified a DMC1 homolog in barley and isolated two independent *dmc1* mutant lines. Analysis of the *dmc1* plants revealed that chromosome bridges, chromosome fragments, micronuclei, and abnormal tetrads were more common during meiosis, compared with the parental variety. Thus, DMC1 is required for DSB repair, crossing-over, and proper chromosome disjunction.

RAD54, a chromatin remodeling factor, forms DNA repair foci in nuclei after DNA damaging treatments. Hirakawa and Matsunaga monitored the RAD54 foci in γ-irradiated *A. thaliana* root cells. The foci were detected in the epidermis, cortex, endodermis, and stem cells in the meristematic zone but not in the quiescent center. The foci were more frequent in the non-S-phase cells. More than half of the foci were attached to the nuclear envelope (NE) in the wild type but the number decreased under INM (Inner Nuclear Membrane)-protein-deficient background, suggesting that the NE plays a role in genome stability.

Plants acquire tolerance to chilling following exposure to low, non-freezing temperatures (cold acclimation). Wang et al. investigated the expression of DNA damage-inducible protein 1 (CsDDI1) and other DDR genes in cold-acclimated cucumber. Inhibition of H_2_O_2_ biosynthesis down-regulated *CsDDI1* gene transcription, suggesting H_2_O_2_ plays a crucial role in triggering cold adaption. CsDDI1 over-expression in *A.thaliana* provides increased tolerance to chilling, lower level of reactive oxygen species, higher catalase and superoxide dismutase activities, and expressions of defense genes. This suggests that the *CsDDI1* gene increases chilling tolerance in plants by enhancing the antioxidant defense system.

The model bryophyte *Physcomitrella patens* exhibits particularly high frequencies of gene targeting, making it an interesting model to study the mechanism of gene targeting. Guyon-Debast et al. reported on the role of the XPF/ERCC1 complex in *P. patents*. Knockout *xpf* and *ercc1* mutants grow under normal condition, however they show high UV-B and MMS (methyl methanesulfonate) sensitivity, indicating that the XPF/ERCC1 complex is involved in the repair of UV- and MMS-induced DNA damage. Using different constructs, they suggest that the complex is required for the homologous recombination between end-out or end-in construct and genome loci. These findings provide clues to improve gene targeting efficiency in other plants.

Aluminum (Al) toxicity is a worldwide problem limiting crop productivity in acidic soils. In *A. thaliana*, Al causes DSBs in roots and ATR, a key factor in DDR, regulates root growth inhibition induced by Al. Szurman-Zubrzycka, Nawrot et al. demonstrated the role of ATR in response to Al toxicity in barley, the most Al-sensitive species among the cereals. They developed barley *atr* mutants, tolerant to Al, in which cell cycle progression was not arrested despite DNA damage accumulation. This knowledge would be useful for growing barley in Al-containing soil.

Nisa et al. reviewed the recent advances in DDR signaling in plants, focusing on the mechanisms leading to cell cycle arrest. They showed that the DDR-triggered cell cycle arrest is induced by SOG1-dependent and SOG1-independent pathways. The former induces cell cycle arrest through several mechanisms, including degradation of the mitotic Cyclin Dependent Kinase B2;1 (CDKB2;1), induction of the CDK inhibitors, and activation of MYB3R repressors. The latter may involve the E2F-RBR1 (RetinoBlastoma Related 1) complexes that function as SOG1 antagonist. They also reviewed recent findings on the relationship between DDR and biotic/abiotic stress responses. Accumulating evidence indicates that DDR is activated in response to pathogen infection or salicylic acid treatment as well as in Al-mediated growth inhibition and chilling stress.

## Author Contributions

AB commented the following articles: Roldán-Arjona et al., Sakamoto et al., Al Khateeb et al., and Waterworth et al.. AS commented the following articles: Szurman-Zubrzycka, Baran et al., Hirakawa and Matsunaga, and Wang et al.. KY commented the following articles: Guyon-Debast et al., Szurman-Zubrzycka, Nawrot et al., and Nisa et al.. All authors and read and revised the complete editorial.

## Conflict of Interest

The authors declare that the research was conducted in the absence of any commercial or financial relationships that could be construed as a potential conflict of interest.

